# Shared and distinct ultra-rare genetic risk for diverse epilepsies: A whole-exome sequencing study of 54,423 individuals across multiple genetic ancestries

**DOI:** 10.1101/2023.02.22.23286310

**Published:** 2023-02-24

**Authors:** Epi25 Collaborative, Siwei Chen, Benjamin M. Neale, Samuel F. Berkovic

**Affiliations:** 1 Analytic and Translational Genetics Unit, Department of Medicine, Massachusetts General Hospital and Harvard Medical School, Boston, MA 02114, USA; 2 Stanley Center for Psychiatric Research, Broad Institute of MIT and Harvard, Cambridge, MA, USA; 3 Program in Medical and Population Genetics, Broad Institute of MIT and Harvard, Cambridge, MA, USA; 4 Epilepsy Research Centre, University of Melbourne, Austin Health, Heidelberg 3084, Australia

## Abstract

Identifying genetic risk factors for highly heterogeneous disorders like epilepsy remains challenging. Here, we present the largest whole-exome sequencing study of epilepsy to date to investigate rare variants that confer risk for a spectrum of epilepsy syndromes. With an unprecedented sample size of >54,000 human exomes, composed of 20,979 deep-phenotyped patients with epilepsy and 33,444 controls, we replicate previous gene findings at exome-wide significance; using a hypothesis-free approach, we identify potential novel associations. Most discoveries are specific to a particular subtype of epilepsy, highlighting distinct genetic contributions to different epilepsies. Combining evidence from rare single nucleotide/short indel-, copy number-, and common variants, we find convergence of different genetic risk factors at the level of individual genes. Further comparing to other exome-sequencing studies, we implicate shared rare variant risk between epilepsy and other neurodevelopmental disorders. Our study also demonstrates the value of collaborative sequencing and deep-phenotyping efforts, which will continue to unravel the complex genetic architecture underlying the heterogeneity of epilepsy.

## Introduction

Epilepsy is a group of heterogeneous disorders, characterized by an enduring predisposition to generate epileptic seizures.^1^ Epilepsy has a prevalence of 4-10 per 1,000 individuals worldwide, making it one of the most common neurological conditions.^2^ The role of genetic contributions to epilepsy causality has been long recognized,^3–5^ yet delineating the full range of genetic causes of the epilepsies remains a core challenge.

Whole-exome sequencing (WES) has proven effective in gene discovery for Mendelian disorders, including familial and severe epilepsy syndromes. There has been an increasing number of genes implicated in the developmental and epileptic encephalopathies (DEEs, [MIM: 308350]), a severe group of epilepsies characterized by early-onset, intractable seizures and developmental delay.^6–10^ In contrast, genes discovered for the milder, more common forms of epilepsies – genetic generalized epilepsy (GGE [MIM: 600669]) and non-acquired focal epilepsy (NAFE [MIM: 604364, 245570]) characterized by generalized and focal seizures, respectively – remain scarce.^1,11–13^ Most discoveries have been based on hypothesis-driven approaches, which are restricted to one or a few predefined candidate gene(s).^12,14^ Hypothesis-free, WES analyses are still in their relative early stages and are not yet powered to produce exome-wide significant results.^11,13,15^ Moreover, most studies have been focused on familial cases and often limited in size; large case collections of common complex epilepsies have only recently been enabled and expanded through global consortia efforts.^11–13,15^

In this study, we present the largest WES analysis of epilepsy to date, from the Epi25 Collaborative, a global collaboration committed to sequencing and deep-phenotyping up to 25K individuals with epilepsy. Our previous data collection and analysis of ~17K and ~29K individuals in case-control cohorts have revealed the extent of rare coding variant risk for all three major types of non-lesional epilepsies (DEEs, GGE, and NAFE). Here, we expand the evaluation to ~54K individuals, comprising 20,979 cases and 33,444 matched controls spanning six genetic ancestries, with improved power for detecting ‘ultra-rare’ variant (URV) association. We apply a hypothesis-free approach to evaluate the excess of URVs (single nucleotide variants [SNVs] and short insertions/deletions [indels]) in cases versus controls, at both an individual-gene and a gene-set level, across the entire exome, and separately for each type of epilepsy. With the enlarged sample size, we discover exome-wide significant genes for different types of epilepsies, implicating both shared and distinct rare variant risk factors. Integrating these findings with associations implicated by copy number variants (CNVs) and genome-wide association study (GWAS), we identify convergence of different types of genetic risk factors in the same genes. More broadly, comparing results to other large-scale WES studies, we provide significant evidence for an overlapping rare variant risk between epilepsy and other neurodevelopmental disorders, although different variant effects may be implicated in a shared gene. Together, our WES analysis at the unprecedented scale makes an important step forward in discovering rare variant risk underlying a spectrum of epilepsy syndromes and offers a valuable resource for generating hypotheses about syndrome-specific etiologies.

## Results

### Study overview

**Extended Data Fig. 1:**
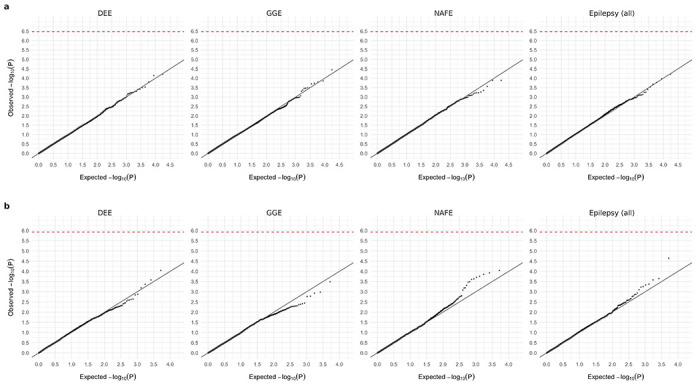
Results from burden analysis of synonymous URVs. **a**,**b**, Burden of synonymous URVs at the individual-gene (**a**) and the gene-set (**b**) level. The observed −log_10_-transformed *P* values are plotted against the expectation given a uniform distribution. Burden analyses are performed across four epilepsy groups – 1,938 DEEs, 5,499 GGE, 9,219 NAFE, and 20,979 epilepsy-affected individuals combined – versus 33,444 controls. *P* values are computed using a Firth logistic regression model with adjustment for sex and ancestry; the red dashed line indicates exome-wide significance *P*=3.4×10^−7^ after Bonferroni correction (see Methods).

We performed WES and harmonized variant detection of an initial dataset of over 70,000 epilepsy-affected and control individuals recruited across 59 sites globally. After stringent quality control (QC; Methods), we included a total of 20,979 individuals with epilepsy and 33,444 controls without known neurological or neuropsychiatric conditions in our URV association analysis, roughly doubling the sample size in our last release of Epi25 WES study.^15^ The samples were predominantly of European genetic ancestries (76.6% non-Finnish and 2.7% Finnish), with smaller proportions of African (7.7%), East Asian (5.3%), South Asian (1.1%), and Admixed American (6.6%) genetic ancestries. Epilepsy cases were matched with controls of the same genetic ancestry as estimated by principal component analysis and samples were pooled for a joint burden analysis of URVs. In the primary analysis, we evaluated the excess of ultra-rare, deleterious SNVs and indels – protein-truncating/damaging missense (MPC [missense badness, PolyPhen-2, and regional constraint]^16^ score≥2) variants observed at no more than five copies among the entire dataset (corresponding to a MAF<0.005%) – in individuals with epilepsy compared to controls, using a Firth logistic regression model with adjustment for sex and genetic ancestry (Methods). We performed the burden analysis at both an individual-gene and a gene-set level, across the entire exome, and separately for each epilepsy type – where 1,938 individuals were diagnosed with DEEs, 5,499 with GGE, and 9,219 with NAFE – as well as for all epilepsy-affected individuals combined (including an additional 4,323 with other epilepsy syndromes). Stringent Bonferroni correction was applied to adjust for 18,531 consensus coding sequence (CCDS) genes and 5,373 gene sets (in the gene-based and gene-set-based burden analysis, respectively) and eight case-control comparisons across four epilepsy groups and two variant classes. To ensure our model was well calibrated, we used ultra-rare synonymous variants as a negative control for all tests (Extended Data Fig. 1). In parallel, we performed CNV discovery and burden analysis on the same dataset (see Methods for details), with a particular focus on the joint burden of rare CNV deletions and protein-truncating URVs.

### Gene-based burden analysis identifies exome-wide significant genes for different types of epilepsies

**Fig. 1:**
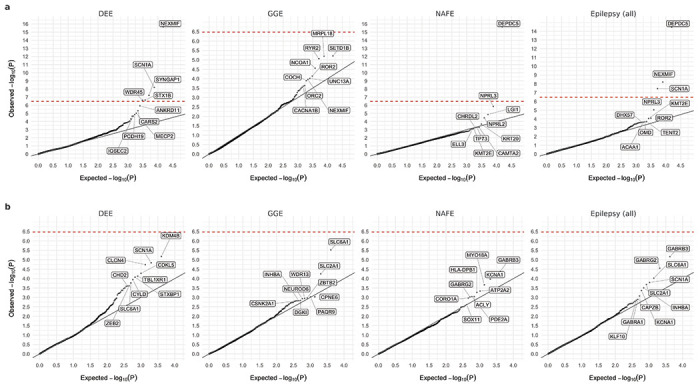
Results from gene-based burden analysis of URVs. **a**,**b**, Burden of protein-truncating (**a**) and damaging missense (**b**) URVs in each protein-coding gene with at least one epilepsy or control carrier. The observed −log_10_-transformed *P* values are plotted against the expectation given a uniform distribution. For each variant class, burden analyses are performed across four epilepsy groups – 1,938 DEEs, 5,499 GGE, 9,219 NAFE, and 20,979 epilepsy-affected individuals combined – versus 33,444 controls. *P* values are computed using a Firth logistic regression model with adjustment for sex and ancestry; the red dashed line indicates exome-wide significance *P*=3.4×10^−7^ after Bonferroni correction (see Methods). Top ten genes with URV burden in epilepsy are labeled.

For gene discovery, we tested the burden of URVs in each protein-coding gene, across all three epilepsy types and all-epilepsy combined (Supplementary Data 1). In the analysis of protein-truncating URVs in DEEs, we identified five genes at exome-wide significance (Fig. 1a; Methods): *NEXMIF* ([MIM: 300524], log[OR]=6.7, *P*<2.2×10^−16^), *SCN1A* ([MIM: 182389], log[OR]=4.1, *P*=6.3×10^−9^), *SYNGAP1* ([MIM: 603384], log[OR]=4.2, *P*=5.9×10^−8^), *STX1B* ([MIM: 601485], log[OR]=4.5, *P*=2.3×10^−7^), and *WDR45* ([MIM: 300526], log[OR]=5.5, *P*=2.4×10^−7^). All five are established epilepsy genes, as reviewed by the GMS Genetic Epilepsy Syndromes panel^17^ with diagnostic level of evidence. *NEXMIF* and *SCN1A* have been consistently the top genes in our prior Epi25 analyses;^13,15^ the other three genes for the first time surpassed the exome-wide significance threshold. The 6^th^ ranked gene – *ANKRD11* ([MIM: 611192]), which approaches exome-wide significance (log[OR]=3.9, *P*=1.2×10^−6^) – emerged as a novel candidate for DEEs. While not directly linked to epilepsy, *ANKRD11* is a known causal gene for the KBG syndrome,^18^ a rare genetic disorder characterized by a range of developmental and neurological abnormalities including epilepsy.^19–21^

Analysis of protein-truncating URVs in NAFE revealed as the most significant gene, *DEPDC5* ([MIM: 614191], log[OR]=2.6, *P*<2.2×10^−16^; Fig. 1a), which encodes part of the GATOR1 complex, a repressor of the mTORC1 pathway that has been prominently associated with focal epilepsies.^14,22–26^ The other two components of the GATOR1 complex, *NPRL3* (MIM: 600928) and *NPRL2* (MIM: 607072), were also among the top associations (ranked at the 2^nd^ and the 4^th^ respectively; *NPRL3:* log[OR]=2.9, *P*=1.4×10^−6^, *NPRL2:* log[OR]=2.8, *P*=3.6×10^−5^), and they together manifested the strongest burden in the subsequent gene-set-based analysis. Noteworthily, *DEPDC5* was the only exome-wide significant hit in the earlier Epi4K WES study of familial NAFE cases;^11^ the expanded inclusion of non-familial cases in our cohort implicates *DEPDC5* in both familial and non-familial settings (log[OR]=3.2 and 2.5, *P*=2.2×10^−9^ and 4.1×10^−15^, respectively; Supplementary Data 2), reinforcing the notion that sporadic and familial forms of epilepsy have shared genetic risk factors.

No genes surpassed the exome-wide significance threshold in GGE. Three genes remained significant when we combined all epilepsy types (Fig. 1a): *DEPDC5* (log[OR]=2.1, *P*=3.4×10^−15^), *NEXMIF* (log[OR]=4.1, *P*=6.3×10^−9^), and *SCN1A* (log[OR]=2.7, *P*=3.5×10^−8^). The signals of enrichment became slightly attenuated compared to the epilepsy type-specific analysis, which may reflect the genetic and etiological heterogeneity of different epilepsies.

In comparison to protein-truncating URVs, burden analysis of damaging missense URVs (MPC ≥2; Methods) did not identify individual genes at exome-wide significance. Nevertheless, the top associations captured known epilepsy genes – notably the *SLC6A1* (MIM: 137165) and *GABRB3* (MIM: 137192) genes, both involved in the GABAergic pathway^13^ and showing enrichment across multiple epilepsy types (Fig. 1b). Most of the previously implicated variants in these two genes were also missense,^27,28^ and our study discovered an additional 24 and 26 damaging missense URVs in *SLC6A1* and *GABRB3*, respectively, increasing the existing candidates by ~50% (Supplementary Data 3). Another top hit – *KDM4B* (MIM: 609765) – was found specifically associated with DEEs, which has not been previously reported.

### Gene-set-based burden analysis facilitates understanding of epilepsy etiology

To further investigate genes and biologically relevant pathways associated with epilepsy, we performed burden tests at a gene-set level, which essentially expanded the test from a single gene to a set of genes that share a particular function. Different from our prior Epi25 analyses, which focused on a few previously implicated gene sets, we systematically tested collections of gene entities that belong to a gene family^29^ or encode a protein complex^30^ (Supplementary Data 4; Methods), in search for novel associations.

**Fig. 2:**
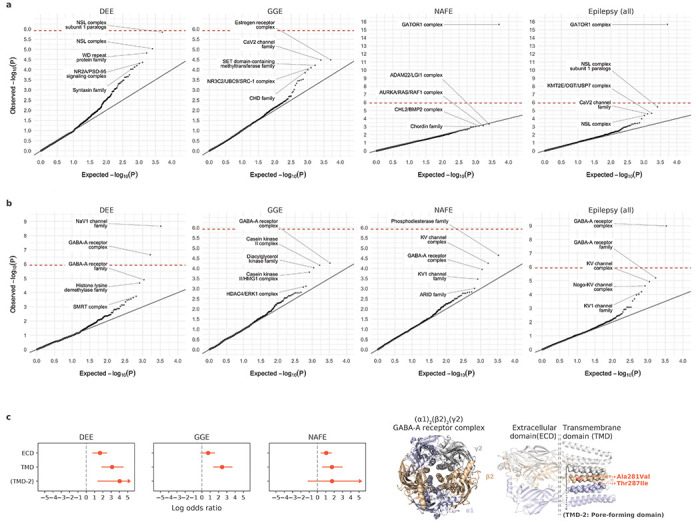
Results from gene-set-based burden analysis of URVs. **a**,**b**, Burden of protein-truncating (**a**) and damaging missense (**b**) URVs in each gene set (gene family/protein complex) with at least one epilepsy or control carrier. The observed −log_10_-transformed *P* values are plotted against the expectation given a uniform distribution. For each variant class, burden analyses are performed across four epilepsy groups – 1,938 DEEs, 5,499 GGE, 9,219 NAFE, and 20,979 epilepsy-affected individuals combined – versus 33,444 controls. *P* values are computed using a Firth logistic regression model with adjustment for sex and ancestry; the red dashed line indicates exome-wide significance *P*=1.2×10^−6^ after Bonferroni correction (see Methods). Top five gene sets with URV burden in epilepsy are labeled. **c**, Burden of damaging missense URVs in the (α1)_2_(β2)_2_(γ2) GABA_A_ receptor complex with respect to its structural domain. Left, forest plots showing the stronger enrichment of damaging missense URVs in the transmembrane domain (TMD) than the extracellular domain (ECD), and the unique signal from DEEs in the second TMD (TMD-2) that forms the ion channel pore. The dot represents the log odds ratio and the bar represents the 95% confidence intervals of the point estimates. Right, a co-crystal structure (PDB ID: 6X3Z) showing the pentameric subunits of the receptor and highlighting the two protein-truncating URVs from DEEs located in the pore-forming domain.

The most pronounced signal, as described in the gene-based burden of protein-truncating URVs, was from the GATOR1 complex in NAFE (log[OR]=2.7, *P*<2.2×10^−16^; Fig. 2a). We identified a total of 56 distinct protein-truncating URVs in the three GATOR1-encoding genes (38 in *DEPDC5*, 11 in *NPRL3*, and 7 in *NPRL2*; Supplementary Data 5), among which 45 appeared novel according to the most recent study of epilepsy-related GATOR1 variants by Baldassari et al.^14^ In contrast to Baldassari et al, where most (>70%) GATOR1 protein-truncating variant carriers were familial, only 20% of the carriers in our study cohort had a known family history of epilepsy. Both familial and non-familial cases showed significant burden of GATOR1 protein-truncating URVs (log[OR]=3.4 and 2.6, *P*=3.7×10^−14^ and *P*<2.2×10^−16^, respectively; Supplementary Data 2), which reinforces the increasingly important role of GATOR1 genes in the etiology of focal epilepsy.

Several strong signals emerged in the analysis of damaging missense URVs, led by well-established ion channel protein complexes and gene families (Fig. 2b). The top association was the GABA_A_ receptor complex, encoded by *GABRA1* (MIM: 137160), *GABRB2* (MIM: 600232), and *GABRG2* (MIM: 137164; [α1]_2_[β2]_2_[γ2], the most abundantly expressed isoform in the brain),^31^ which controls the majority of inhibitory signaling in the central nervous system. The complex showed extensive enrichment across all epilepsy types (DEEs: log[OR]=2.0, *P*=2.2×10^−7^, GGE: log[OR]=1.3, *P*=5.5×10^−5^, NAFE: log[OR]=1.1, *P*=1.1×10^−4^), recapturing the pervasive role of GABA_A_ receptors across the spectrum of severities in epilepsy (reviewed in 32). Further dissecting the signals with respect to the structural domain of the complex, we observed stronger enrichment in the transmembrane domains (TMDs) than the extracellular domain (ECD), particularly for DEEs and GGE; and DEEs presented a unique signal in the second TM *α*-helix lining the ion channel pore of the receptor^33^ (Fig. 2c; Supplementary Data 6). To avoid potential bias introduced by MPC prioritizing domains of regional missense constraint, we recapitulated the results using other missense deleteriousness metrics (PolyPhen-2^34^ and SIFT^35^; Supplementary Data 6). These patterns collectively point to an association of damaging missense URVs in the pore-forming domain with a more severe form of epilepsy.

Potential novel associations were found in two gene sets: the NSL complex (with protein-truncating URVs in *KANSL1* [MIM: 612452], *KANSL2* [MIM: 615488], and *PHF20* [MIM: 610335]) and the phosphodiesterase (PDE) gene family (with damaging missense URVs in *PDE2A* [MIM: 602658] and *PDE10A* [MIM: 610652]), associated with DEEs and NAFE, respectively. Despite the sparsity of URVs, our results broaden the potential allelic spectrum of variants that may confer risk to different types of epilepsies.

### Protein structural analysis characterizes missense URVs in ion channel genes

**Fig. 3:**
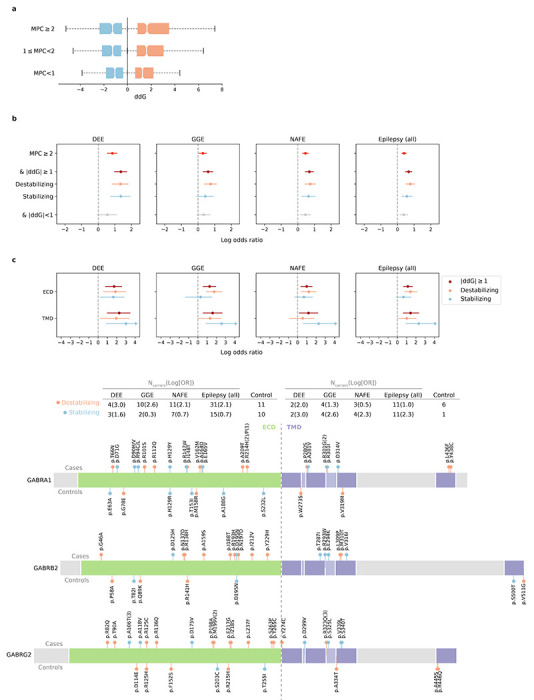
Protein structural analysis of missense URVs in ion channel genes. **a**, Correlation between ddG and MPC in measuring the deleteriousness of missense URVs. ddG values are computed for 1,782 missense URVs across 16 ion channel protein complexes (see Methods). A higher absolute ddG value suggests a more deleterious effect on protein stability; positive (orange) and negative (blue) values suggest destabilizing and stabilizing effects, respectively. **b**, Burden of damaging missense URVs stratified by ddG. Stronger enrichment is observed when applying |ddG|>1 to further prioritize damaging missense URVs with MPC≥2. **c**, Burden and distribution of destabilizing (ddG≥1) and stabilizing (ddG≤−1) missense URVs on the (α1)_2_(β2)_2_(γ2) GABA_A_ receptor complex with respect to its structural domain. Top, forest plots showing the stronger enrichment of destabilizing missense URVs (orange) in the extracellular domain (ECD) and stabilizing missense URVs (blue) in the transmembrane domain (TMD). Bottom, schematic plots displaying the distribution of destabilizing and stabilizing missense URVs on GABA_A_ receptor proteins. URVs found in epilepsy cases are plotted above the protein and those from controls are plotted below the protein. The number of epilepsy and control carriers are listed in the table above. In the forest plots in **c** and **d**, the dot represents the log odds ratio and the bar represents the 95% confidence intervals of the point estimates.

The strong burden of damaging missense, but not protein-truncating, URVs in genes encoding ion channels suggests a pathophysiological mechanism of protein alteration (e.g., gain-of-function or dominant-negative effects) rather than haploinsufficiency. Given the specialized structure of ion channels, we sought to further characterize missense URVs at a protein structure level. Specifically, we leveraged experimentally resolved three-dimensional structures of ion channel proteins, most of which were co-crystallized heterotrimeric subunits, and applied Rosetta^36^ to assess the energy changes (change in Gibbs free energy ΔΔG/ddG in abbreviation) of protein folding upon a particular missense URV; a decrease in Gibbs free energy of unfolding, i.e., a positive ddG value suggests a destabilizing effect of the variant on protein and a negative value suggests a stabilizing effect. We computed ddG for a total of 1,782 missense URVs – independent of MPC deleteriousness – across 16 ion channel protein complexes (Supplementary Data 7; Methods). There was, as expected, a positive correlation between ddG and MPC (*ρ*=0.15, *P*=8.3×10^−11^; Fig. 3a), indicating that missense URVs in missense-constrained regions are more likely to cause a change in protein stability.

Even with MPC being a strong indicator of damaging missense burden in epilepsies, incorporating ddG further stratified the association signals (Fig. 3b; Supplementary Data 8). Significant enrichment was found for both destabilizing (ddG≥1 kcal/mol) and stabilizing (ddG≤−1 kcal/mol) URVs, which suggests a diverging molecular basis for these missense URVs. To explore potential structural properties that are associated with the protein stability change, we again dissected the signals by the protein structural domains. Divergent distributions were found for destabilizing and stabilizing missense URVs, with the former enriched in the ECD of the complex and the latter in the TMD (Fig. 3c; Supplementary Data 9). While only functional tests can further elucidate the underlying molecular mechanisms, our analyses provide a set of missense URVs to test in epilepsy model systems, which might reveal the variable effects on protein function such mutations are creating and add to the explanation of how ion channel dysfunction could produce a broad spectrum of epilepsy syndromes.

### Burden of CNV deletions converges with protein-truncating URVs

In parallel with SNVs and indels, we performed variant calling of CNVs on the same dataset (Methods). After sample QC, we examined gene burden of rare CNVs in 18,963 epilepsy cases - including 1,743 DEEs, 4,980 GGE, and 8,425 NAFE – versus 29,804 controls (~90% of initial; Methods). A gene was considered affected by a CNV if ≥10% of its coding exons were deleted or ≥75% were duplicated.

**Fig. 4:**
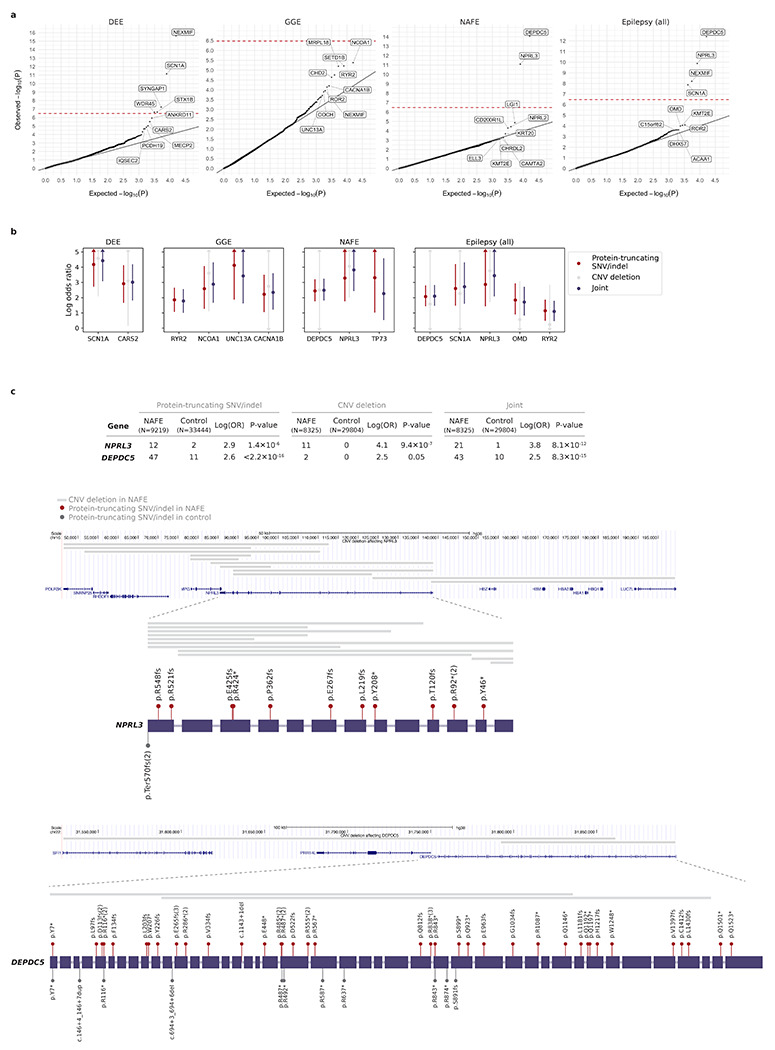
Convergence of CNV deletions and protein-truncating URVs in gene-based burden. **a**, Joint burden of CNV deletions and protein-truncating URVs in each protein-coding gene with at least one epilepsy or control carrier. The observed −log_10_-transformed *P* values are plotted against the expectation given a uniform distribution. Joint burden analyses are performed on the subset of samples that passed CNV calling QC (see Methods), across four epilepsy groups – 1,743 DEEs, 4,980 GGE, 8,425 NAFE, and 18,963 epilepsy-affected individuals combined – versus 29,804controls; for genes that do not have a CNV deletion called, results from the burden analysis of protein-truncating URVs on the full sample set are shown. *P* values are computed using a Firth logistic regression model with adjustment for sex and ancestry; the red dashed line indicates exome-wide significance *P*=3.4×10^−7^ after Bonferroni correction (see Methods). Top ten genes with variant burden in epilepsy are labeled. **b**, Joint burden of CNV deletions and protein-truncating URVs in the top ten genes ranked by protein-truncating URV burden. For comparison, the burden of protein-truncating URVs (SNVs/indels; red), CNV deletions (gray), and the joint (purple) are analyzed on the same sample subset as described in **a**. The dot represents the log odds ratio and the bar represents the 95% confidence intervals of the point estimates; only enrichment in epilepsy (log odds ratio>0) are shown. **c**, Genomic location and distribution of CNV deletions and protein-truncating URVs with respect to the *NPRL3* and *DEPDC5* genes. Variants found in epilepsy cases (red) are plotted above the schematic gene plots and those from controls (gray) are plotted below the gene. The number of epilepsy and control carriers are listed in the table above.

The most significant signal was from CNV deletions in the *NPRL3* gene, with 11 deletions found in NAFE cases versus 0 in controls (log[OR]=4.1, *P*=9.4×10^−7^; Supplementary Data 10). Notably, *NPRL3* was also one of the top hits implicated by protein-truncating URVs in NAFE, and jointly analyzing the two rendered *NPRL3* exome-wide significant (log[OR]=3.8, *P*=8.1×10^−12^; Fig. 4a and Supplementary Data 10). Among the top ten genes with protein-truncating URV burden, about one-third (14/[10×4 epilepsy groups]) were found affected by a CNV deletion, and the vast majority (11/14) showed enrichment in epilepsy cases (log[OR]>0; Fig. 4b). These included *DEPDC5*, which together with *NPRL3* reinforces a haploinsufficiency mechanism for GATOR1-related focal epilepsies (Fig. 4c). Strengthened burden was also found for potential novel genes – e.g., *CARS2* (MIM: 612800) in DEEs and *NCOA1* (MIM: 602691) in GGE, both with accumulating evidence from literature and case reports.^37–43^ Analysis of CNV duplications did not show any individual genes close to exome-wide significance (Supplementary Data 10). Collectively, the joint burden analysis suggests at least partial convergence in the protein-truncating- effect caused by SNVs/indels and CNVs, and therefore, it may provide a strategy for improving the power of detecting rare, large-effect genetic risk factors for epilepsy.

### Burden of URVs reveals shared genetic risk between common and rare variation for GGE

**Fig. 5:**
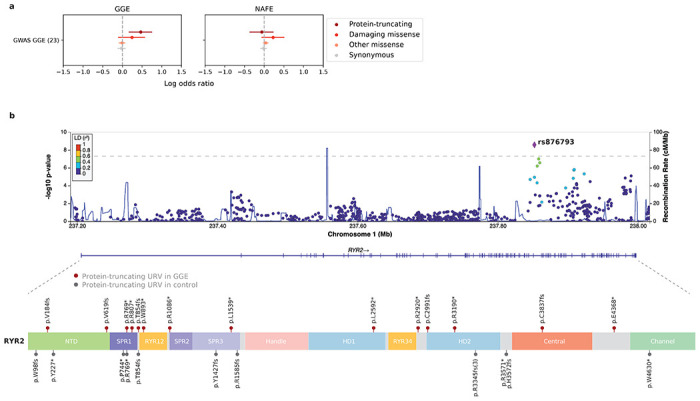
Shared genetic risk between common and rare variation for GGE. **a**, Burden of URVs in genes implicated by GGE GWAS loci. Burden analyses are performed across four variant classes (colored by the inferred consequence) and two epilepsy groups – 5,499 GGE and 9,219 NAFE – versus 33,444 controls. Significant enrichment is only observed for protein-truncating URVs from GGE but not any from NAFE. The dot represents the log odds ratio and the bar represents the 95% confidence intervals of the point estimates. **b**, Genomic location and distribution of common variant (GWAS) association and protein-truncating URVs on the *RYR2* gene. Top, a LocusZoom plot displaying the GGE GWAS hit (rs876793) located in the intron of *RYR2*. Bottom, a schematic protein plot displaying the distribution of protein-truncating URVs on RYR2. URVs found in epilepsy cases (red) are plotted above the protein and those from controls (gray) are plotted below the protein.

Similar to other common neurodevelopmental disorders, the common forms of epilepsy – GGE and NAFE – have both common and rare genetic risk factors. In partnership with the International League Against Epilepsy (ILAE) consortium, we performed GWAS meta-analysis of over 29K individuals with common epilepsies,^44^ which revealed 26 genome-wide significant loci with markedly different genetic architectures between GGE (22 loci) and NAFE (0 loci). To investigate the overlap of epilepsy association between common and rare variation, we tested the burden of URVs in 23 genes that were prioritized as the likely causal genes underlying the 22 GGE loci. The analysis identified significant enrichment for protein-truncating URVs from GGE in the 23 GWAS genes, while in contrast, none for URVs from NAFE (Fig. 5a; Supplementary Data 11). This result has two-fold implication: first, there is emerging evidence of convergent common and rare variant risk in the same genes for epilepsy, and second, the convergence tends to be epilepsy type-specific.

At the individual gene level, 13 of the 23 GGE GWAS genes showed an excess of protein-truncating URVs (log[OR] 0.2-2.6; Supplementary Data 11). The lead gene was *RYR2* (MIM: 180902), in which 14 protein-truncating URVs were observed in our GGE cohort (log[OR]=1.8, *P*=8.6×10^−5^), and the reported GWAS hit was located in the intronic region (rs876793; Fig. 5b). *RYR2* encodes a ryanodine receptor that mediates the release of Ca(2+) from endoplasmic/sarcoplasmic reticulum into cytoplasm for excitation-contraction coupling. Mutations in *RYR2* have been well-known in the etiology of arrhythmogenic disorders,^45–50^ while more recent studies reported that the same mutation can cause GGE independent of arrhythmias.^51,52^ Our finding, together with the GWAS result, adds weight to the hypothesis that *RYR2* mutations likely constitute a neuro-cardiac calcium channelopathy,^51,52^ where mutant receptors may induce either arrhythmias or GGE depending on their selective expression in the heart or in the brain.

### Burden analysis implicates shared rare variant risk between epilepsy and other neurodevelopmental disorders

**Fig. 6:**
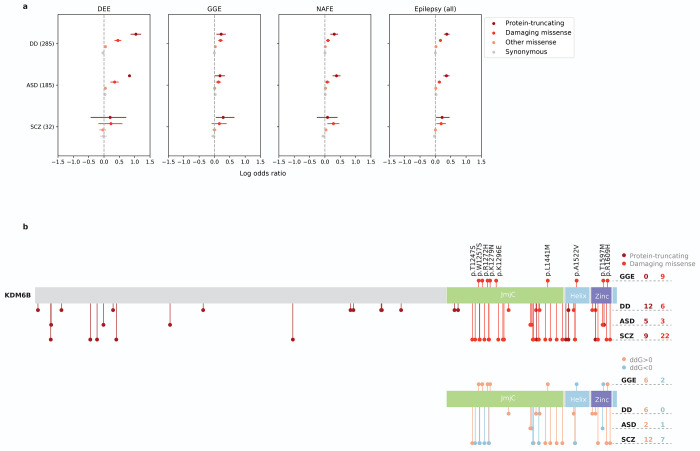
Shared rare variant risk between epilepsy and other NDDs. **a**, Burden of URVs in genes implicated by WES of severe developmental disorders (DD), autism spectrum disorder (ASD), and schizophrenia (SCZ). Burden analyses are performed across four variant classes (colored by the inferred consequence) and four epilepsy groups – 1,938 DEEs, 5,499 GGE, 9,219 NAFE, and 20,979 epilepsy-affected individuals combined – versus 33,444 controls. Overall, DD/ASD-associated genes show stronger enrichment of epilepsy URVs than SCZ. **b**, Distribution of rare variants from GGE and other NDDs on the *KDM6B* gene. Top, a schematic protein plot displaying the distribution of protein-truncating (darker red) and damaging missense (lighter red) variants on KDM6B. Bottom, a schematic protein plot displaying the distribution of damaging missense variants with a likely destabilizing (ddG>0; orange) and stabilizing (ddG<0; blue) effect on KDM6B. In both plots, variants found in GGE are plotted above the protein and those from other NDDs are plotted below the protein (in the order of DD, ASD, and SCZ as labeled); the number of variant carriers are listed accordingly on the right.

Recent WES studies have revealed substantial rare variant risk for neurodevelopmental disorders (NDDs). Analysis of *de novo* mutations in severe developmental disorders (DDs) has discovered 285 genes at exome-wide significance,^53^ and more recent rare variant associations in autism spectrum disorder (ASD)^54^ and schizophrenia (SCZ)^55^ have implicated 185 and 32 genes at a false discovery rate of 5%, respectively. To explore how these and our findings may point to common genetic etiologies, we examined the burden of URVs from epilepsy in the established NDD genes (Supplementary Data 12). Significant enrichment was found for all three gene sets associated with DD, ASD, and SCZ (Fig. 6a), suggesting that there is shared genetic risk of rare variation among the broader spectrum of NDDs. DD and ASD presented stronger signals than SCZ, across all epilepsy types (being strongest in DEEs) and for both protein-truncating and damaging missense URVs. This pattern implies a larger overlapping genetic component between epilepsy and DD/ASD than SCZ, which is in line with the high comorbidity of DD/ASD and epilepsy, in particular DEEs. Meanwhile, given the known genetic overlapping between DD and ASD, we repeated the analyses on the subsets of mutually exclusive NDD genes (i.e., 196 DD-only, 99 ASD-only, and 22 SCZ-only genes, respectively). Although attenuated, there remained clear rare variant signals shared by epilepsy and other NDDs (Supplementary Data 12).

About one-third (136/409) NDD genes showed nominally significant enrichment of deleterious URVs in at least one epilepsy type (Supplementary Data 12). The vast majority (128/136=94.1%) were DD/ASD genes, and only one gene – *KDM6B* (MIM: 611577) – was shared by all three NDD gene sets. *KDM6B* encodes a lysine-specific demethylase that has been recognized as a critical player in neurogenesis and neuronal cell-type diversification.^56–60^ Interestingly, URVs in *KDM6B* associated with epilepsy were exclusively missense (MPC≥2), whereas *KDM6B* variants implicated in DDs were predominately protein-truncating (Fig. 6b; Supplementary Data 13). All missense variants were clustered at the KDM6B catalytic domain (JmjC) and C-terminal helix/zinc motifs, which are important for enzyme-cofactor binding and protein stability.^61^ Protein structural analysis predicted that most of the damaging missense variants tend to have a destabilizing effect on the KDM6B protein (ddG>0; Supplementary Data 13), especially those in DDs, while diverging effects were observed for epilepsy and SCZ (Fig. 6b). These results suggest that, even converging in the same gene, rare variant risk may differ in its severity and/or the molecular mechanism that underlies specific phenotypes of NDDs.

## Discussion

In the largest WES study of epilepsies to date, we characterize the contribution of ultra-rare genetic variation to a severity spectrum of epilepsy syndromes. This work, from the Epi25 Collaborative, involves global efforts in aggregating sequence data, deep-phenotyping epilepsy cohorts, harmonizing variant detection and quality control, and finally analyzing and interpreting the genetic data for etiological and clinical implications.

**Extended Data Fig. 2:**
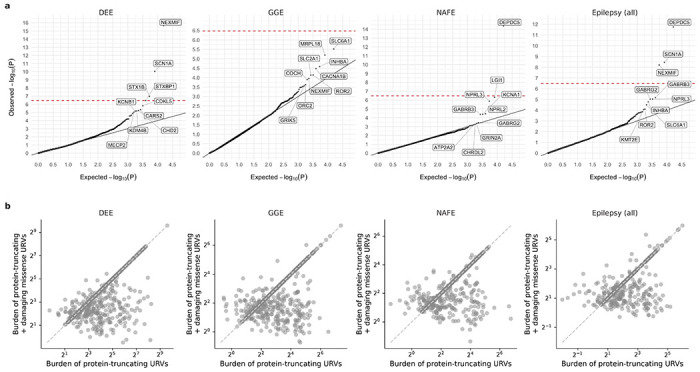
Results from burden analysis of protein-truncating and damaging missense URVs combined. a, Joint burden of protein-truncating and damaging missense URVs at the individual-gene level. The observed −log10-transformed P values are plotted against the expectation given a uniform distribution. Burden analyses are performed across four epilepsy groups – 1,938 DEEs, 5,499 GGE, 9,219 NAFE, and 20,979 epilepsy-affected individuals combined – versus 33,444 controls. P values are computed using a Firth logistic regression model with adjustment for sex and ancestry; the red dashed line indicates exome-wide significance P=3.4×10-7 after Bonferroni correction (see Methods). b, Comparison of the joint burden in **a** with the burden of protein-truncating URVs. The odds ratio (OR) of protein-truncating plus damaging missense URVs (y-axis) and that of protein-truncating URVs alone (x-axis) are compared. Each dot represents a gene with nominally significant enrichment (OR>0 and P<0.05) of either protein-truncating URVs or the two variant classes combined.

Our exome-wide burden analyses redemonstrated the role of known epilepsy genes with improved power and discovered potential novel rare variant risk factors for different types of epilepsies. Most associations were identified in a particular epilepsy type, implicating distinct genetic etiologies underlying different epilepsies. Protein-truncating URVs presented the strongest signal, with six individual genes surpassing the stringent exome-wide significance threshold. Five genes (*NEXMIF, SCN1A, SYNGAP1, STX1B*, and *WDR45*) were associated with the severe group of DEEs, while notably, the most significantly-associated gene – *DEPDC5* – was found in NAFE. The implication of GATOR1 with the enlarged sample size has particular clinical applications – given that GATOR1 functions as a negative regulator of the mTORC1 pathway, mTORC1 inhibitors may offer a promising treatment strategy for patients carrying deleterious GATOR1 variants.^62^ In comparison to protein-truncating URVs, analysis of damaging missense URVs remained underpowered to identify individual genes at exome-wide significance. Yet, strong associations emerged when aggregating sets of genes that share common functions. The top associations were predominantly genes encoding ion channel complexes, such as Nav/Kv channels and GABA_A_ receptors. These gene sets did not show significant enrichment of protein-truncating URVs, which suggests a more diverse pathophysiological mechanism than haploinsufficiency. We further explored this through protein structural analysis and indeed observed diverging effects of missense URVs on ion channel protein stability. In particular, the enrichment of stabilizing URVs in the pore-forming domain for inhibitory neurotransmission appeared intriguing given that the pathophysiological condition of epilepsy is hyperexcitability. One hypothesis fitting with functional studies in channelopathies is that the over-stabilization of a particular structural conformation would interfere with the conformational dynamics required for ion channel gating – for instance, recent structural studies have established a ‘dual-gate’ model:^63^ upon sustained agonist binding the ion channel will gradually transit from the active agonist-bound conformation to an agonist-bound shut state refractory to activation (i.e., desensitization); mutant ion channels favoring a desensitized conformation may consequently reduce the efficacy of GABAergic inhibition and lead to an elevated excitability. As both loss- and gain-of-function mechanisms underlying conformational changes in ligand/voltage-gated ion channels being increasingly described in rare epilepsies with distinguishable clinical features,^28,64–66^ our results may add to the molecular mechanisms that explain the varying types of epilepsies associated with ion channel dysfunction. Meanwhile, we emphasize the necessity of dedicated functional investigation for specific missense variants. In this study, we deliberately separated the analysis of protein-truncating and damaging missense URVs with a view to delineating the differing mechanisms; assuming a protein-truncating-like effect for all damaging missense URVs identified no additional significant genes but weakened our analytical power – most (~90%) genes enriched for protein-truncating URVs had either no damaging missense URV or decreased enrichment when the two variant classes were combined (Extended Data Fig. 2).

Potential novel associations were identified or strengthened in several genes and gene sets. Top candidates were predominately implicated in DEEs, including *ANKRD11* gene and the NSL complex with protein-truncating URVs and *KDM4B* gene with damaging missense URVs. Numerous experiments have demonstrated the importance of NSL complex in regulating core transcriptional and signaling networks required for normal development (reviewed in ^67^), and mutations or deregulation of NSL complex genes has been associated with neurodevelopmental disorders.^67^ Haploinsufficiency of *KANSL1*, for instance, is a known monogenic cause of the KdVS syndrome,^68,69^ a multisystem disorder commonly accompanied by epileptic seizures.^70–72^ The *KDM4B* gene encodes a demethylase enzyme that regulates gene expression in the brain from embryonic stages.^73^ Neuron-specific kdm4b-deficient mice were shown to display spontaneous epileptic-like seizures, and more recent data implicated *KDM4B* rare variants in global developmental delay.^74^ Collectively, these genes have an already established role in neurodevelopmental disorders that present shared clinical characteristics with DEEs. This clinical overlap lends support to these newly implicated associations, while requiring advanced understanding of the crosstalk between epilepsy and developmental encephalopathies within DEEs (e.g. for a specific case, whether developmental encephalopathy is a direct sequential consequence of epileptic seizures or, they share a common genetic etiology but different pathological pathways and occur in parallel [reviewed in ^75^]). Such complexity was also reflected by the substantial excess of DEEs-URVs in DD-associated genes. Another new candidate – the PDE gene family – was found associated with NAFE. PDE enzymes catalyze the hydrolysis of cAMP and cGMP, two key second messengers modulating a variety of neuronal pathways^76–78^, in particular with a dual regulatory function to increase the strength of excitatory neural circuits and decrease inhibitory synaptic plasticity;^79,80^ it is thus plausible that loss of PDE catalytic activity may result in a net excess of neural excitation and an increased susceptibility to epilepsy. In support of this, previous studies have reported that administration of PDE10A inhibitors induced epileptic seizures.^81,82^ Lastly, a particularly noteworthy finding was the *RYR2* gene associated with GGE, which was prioritized from combining evidence of rare and common genetic variation. This result provides an example of convergent epilepsy generic risk across the allele frequency spectrum and also represents an instance of epilepsy subtype-specific association, motivating the generation of more specific mechanistic hypotheses. While we only highlighted GGE in the present analysis, we note that we have previously observed the effect in both GGE and NAFE, using a relatively lenient inclusion criterion (by aggregating URVs across the top 100 genes from GWAS).^83^ Together, we would suggest that the convergence of rare and common variant risk may also exist in NAFE, though being much stronger in GGE.

**Extended Data Fig. 3:**
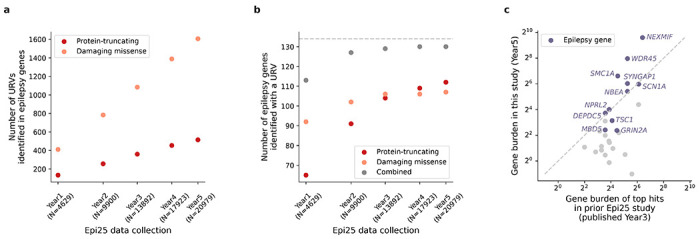
URV discovery and burden results across Epi25 data collection. **a**, Increase in the number of protein-truncating and damaging missense URVs discovered in epilepsy genes with a known monogenic cause. **b**, Increase in the number of monogenic epilepsy genes identified with a protein-truncating or damaging missense URV. In **a** and **b**, variant/gene count is plotted against the year of Epi25 data collection; the total number of epilepsy cases analyzed in each year is indicated in parenthesis. **c**, URV burden of previously top-ranked genes in this study. The odds ratio of protein-truncating URVs in genes from this study (y-axis) and the prior Epi25 publication (x-axis) are compared. Each dot represents one of the top ten genes implicated by our previous burden analysis (across three epilepsy types). Genes with a known monogenic/X-linked cause are labeled and colored in purple.

Besides nominating new genes, identifying new candidate variants in known epilepsy genes will also facilitate the characterization of specific mechanisms, especially given the highly heterogeneous nature of epilepsy. Over the past five-year efforts from Epi25 WES, there has been a steady increase in the number of deleterious URVs discovered in epilepsy-associated genes (Extended Data Fig. 3a); almost all (130/134) genes with a known monogenic cause have been identified with at least one deleterious URVs (Extended Data Fig. 3b), providing a valuable resource for downstream functional analysis. Interestingly, while the number of damaging missense URVs increases at a higher rate than protein-truncating URVs, the number of additional genes identified with a missense URV grows more slowly (Extended Data Fig. 3b). Such a pattern reflects an accumulation of candidate missense URVs in the same set of genes, which highlights the need of effective approaches to characterize and/or categorize the function of missense variants. This has become particularly important as it is increasingly recognized that the variant functional category can correspond not only to patients’ clinical phenotypes but also to their response to treatment.^64,84–88^

The global collaborative efforts of large-scale sequencing and deep-phenotyping have been gaining power to discover ultra-rare genetic risk factors underlying specific epilepsy syndromes. Compared to our prior URV results,^15^ the top genes that maintained or obtained stronger association in this enlarged study are all known epilepsy genes (Extended Data Fig. 3c). This trend demonstrates a high replicability of existing gene findings, and likewise, calls for larger sample sizes to confirm the present results. Substantial sample sizes will be needed for the common complex forms of epilepsies; as projected in our initial WES study,^13^ with >9,000 cases and >20,000 controls we now begin to identify the first exome-wide significant gene for NAFE. The challenge comes from both the heterogeneity in the electroclinical syndromes within each epilepsy subtype (e.g., childhood/juvenile absence epilepsy and juvenile myoclonic epilepsy in GGE) and the heterogeneity in their genetic etiologies, for which there is inevitably a compromise between larger sample size and finer sample classification. A promising strategy to accelerate gene discovery is to integrate results of URVs with other types of genetic variation (e.g., CNVs, common variants); as there is growing evidence that different genetic risk factors converge at least partially in the same genes, an extended model that jointly analyzes these variants would likely provide the most powerful and informative results beyond any single approach. Overall, the ongoing sequencing and genotyping efforts, together with the ever-increasing scale of genetic association studies, will continue to expand and/or refine our understanding of the genetic architecture of epilepsy, continue to delineate specific underlying pathophysiological processes, and hopefully enable a move towards more targeted treatment approaches through both precision diagnosis and the development of precision, or gene-based, therapies.

## Methods

### Study design and participants

We collected DNA and detailed phenotyping data of individuals with epilepsy from 59 participating Epi25 sites in Europe, North America, Australasia, and Asia (Supplementary Information). In total, we analyzed 20,979 epilepsy cases – including 1,938, 5,499, and 9,219 individuals with DDEs, GGE, and NAFE, respectively, and 4,323 with other epilepsies (mostly lesional focal epilepsy [2,495] and febrile seizures [FS]/FS+ [327]) – and 33,444 controls. Control individuals were aggregated from a subset of Epi25 sites, local collections at the Broad Institute, or dbGaP and were not screened for neurological or neuropsychiatric conditions (see Supplementary Table 2).

### Phenotyping procedures

Epilepsies were diagnosed by epileptologists on clinical grounds (see below for specific criteria for DEEs, GGE, and NAFE) in accordance with the International League Against Epilepsy (ILAE) classification at the time of diagnosis and recruitment.^1,13,15^ Phenotyping data were entered into the Epi25 Data repository (https://github.com/Epi25/epi25-edc) via case record forms hosted on the REDCap platform^89^. The data fields do not contain protected health information (PHI). Data collected from previous coordinated efforts with phenotyping on databases (e.g., the Epilepsy Phenome/Genome Project^90^ and the EpiPGX project [Web Resources]) were integrated via scripted transformations. All phenotyping data underwent review for uniformity among sites and quality control (QC) by automated data checking and manual review as required; the process was overseen by a phenotyping committee with clinical expertise.

### Epilepsy case definitions

Epilepsy diagnoses and classification for Epi25 have been described previously.^13,15^ In brief, diagnosis of DEEs required severe refractory epilepsy of unknown etiology, with developmental plateau or regression, and with epileptiform features on electroencephalogram (EEG). Diagnosis of GGE required a history of generalized seizure types (generalized tonic-clonic, absence, or myoclonic seizures) with generalized epileptiform discharges on EEG; exclusion criteria included focal seizures, moderate-to-severe intellectual disability, and epileptogenic lesions on neuroimaging if available. Diagnosis of NAFE required a history of focal seizures with either focal epileptiform discharges or normal findings on EEG; exclusion criteria included primary generalized seizures, moderate-to-severe intellectual disability, and neuroimaging lesions (except hippocampal sclerosis).

### Informed consent

Adult participants, or the legal guardian of child participants, provided signed informed consent at participating centers based on the local ethical requirements at the time of collection. The consent was required not to exclude data sharing to be included in the study. Consent forms for samples collected after January 25, 2015 required specific language according to the National Institutes of Health’s Genomic Data Sharing Policy (see web resources).

### Whole-exome sequencing data generation

All samples were sequenced at the Broad Institute of MIT and Harvard on the Illumina HiSeq X or NovaSeq 6000 platforms with 150 bp paired-end reads. Exome capture was performed using multiple kits: the Illumina Nextera Rapid Capture Exomes or TruSeq Rapid Exome enrichment kit (target size 38 Mb) and the Twist Custom Capture (target size 37 Mb). Sequence data in the form of BAM files were generated via the Picard data-processing pipeline and well-calibrated reads were aligned to the human reference GRCh38. Variants were jointly called across all samples via the Genome Analysis Toolkit (GATK) best-practice pipeline^91^ and were annotated using Variant Effect Predictor (VEP)^92^ with custom annotations, including LOFTEE (Loss-Of-Function Transcript Effect Estimator)^93^ and MPC (missense badness, PolyPhen-2, and regional constraint),^16^ using Hail.^94^

### Variant and sample QC

Initial variant QC criteria included: (1) genotype quality (GQ) ≥20, (2) read depth (DP) ≥20, (3) allele balance (AB) ≥0.2 and ≤0.8, (4) passing the GATK Variant Quality Score Recalibration (VQSR) filter, (5) residing in GENCODE coding regions that were well-covered by both capture platforms, where 80% of the Illumina or Twist sequenced samples had at least 10x coverage, and (6) outside of the low-complexity (LCR) regions.^95^ Additional variant QC were applied after sample QC (see below for details): (1) call rate ≥0.98, (2) case-control call rate difference ≤0.02, and (3) Hardy-Weinberg Equilibrium (HWE) test p value ≥10^−6^.

Sample QC criteria, on the basis of all sequenced samples and the initial QC-ed variants, included: (1) mean call rate ≥0.90, (2) mean GQ ≥57, (3) mean DP ≥25, (4) freemix contamination estimate ≤2.5%, (5) percent chimeric reads ≤2%, and (6) the genetically imputed sex matching with self-reported sex. We performed principal component analysis (PCA) to classify samples into genetic ancestral groups, using a random forest model trained on the 1000 Genomes data; samples with a probability ≥0.9 to be one of the six populations – Non-Finnish European (NFE), Finnish (FIN), African (AFR), East Asian (EAS), South Asian (SAS), Ad Mixed American (AMR) – were retained. Within each ancestral group, we examined cryptic relatedness based on identity-by-descent (IBD) estimates and excluded one sample from each pair of related individuals with an IBD>0.2. Additional sample QC were applied on a population- and cohort-specific basis, which excluded outliers with >4 standard deviations from the mean of (1) transition/transversion ratio, (2) heterozygous/homozygous ratio, and (3) insertion/deletion ratio. To control for residual population stratification, we further excluded samples and/or cohorts that show extreme counts of synonymous singletons. The number of samples passed QC at each step is detailed in Supplementary Information.

### Exome-wide burden analysis

To evaluate the excess of rare, deleterious protein-coding variants in individuals with epilepsy, we performed burden analysis across the entire exome, at both an individual-gene and a gene-set level. “Ultra-rare” variants (URVs) were defined as variants observed no more than five copies among the combined case-control cohort, which corresponded to a minor allele frequency (MAF) <0.005%. Deleterious variants were defined and categorized into two classes: (1) protein-truncating annotated by LOFTEE and (2) damaging missense with an MPC score ≥2. We tested the burden of each URV class by regressing the case-control status on the URVs aggregated across a target gene or gene set in an individual, using a Firth regression model adjusting for sex and ancestry (the PCA-predicted genetic ancestral group and the top ten PCs). We further included the exome-wide count of synonymous singletons as an additional covariate to better control for residual population stratification not captured by PCs.^13^

We performed the burden analyses for each of the three major epilepsy types – DEEs, GGE, and NAFE – and for all epilepsy-affected individuals combined. At the individual-gene level, we tested all protein-coding genes with at least one epilepsy or control carrier (protein-truncating: N=15,083, 15,236, 15,398, and 15,903 for the analysis of DEEs, GGE, NAFE, and all-epilepsy combined, respectively; damaging missense: N=4,013, 4,057, 4,105, and 4,194; synonymous: N=17,460, 17,463, 17,465, and 17,472). At the gene-set level, we tested collections of gene entities that belong to the same gene family^29^ or encode a particular protein complex^30^ and have at least one epilepsy or control carrier (protein-truncating: N=5,080, 5,070, 5,091, and 5,126 for the analysis of DEEs, GGE, NAFE, and all-epilepsy combined, respectively; damaging missense: N=3,256, 3,279, 3,298, and 3,343; synonymous: N=5,209). Exome-wide significance was determined by Bonferroni correction accounting for 18,531 consensus coding sequence (CCDS) genes or 5,373 gene sets – across four epilepsy groups and two variant classes – at *P*=3.4×10^−7^ and *P*=1.2×10^−6^ for the gene- and gene-set-based burden analysis, respectively.

### Protein structural analysis

We applied a metric^36^ that assesses the change in Gibbs free energy (ΔΔG/ddG in abbreviation) of protein folding induced by a mutation to characterize missense URVs identified in ion channel genes. In total, we computed ddG for 1,782 missense URVs on 16 ion channel protein complexes with experimentally resolved three-dimensional structures available (Supplementary Data 7). A positive ddG value suggests a decrease in Gibbs free energy of protein unfolding, i.e., a destabilizing effect of the mutation on protein, and a negative ddG value suggests a stabilizing effect. In the relevant burden analysis, we used |ddG|≥1 kcal/mol to prioritize variants that are likely to cause a change in protein stability.

### Copy number variant (CNV) calling and burden analysis

To call CNVs from the raw exome data, GATK-gCNV^96^ was used. In brief, GATK-gCNV is a Bayesian CNV caller, which adjusts for biases (i.e. GC content) introduced through capture kits and sequencing, while simultaneously accounting for systematic and technical differences. The raw sequencing files were compressed into counts and used as input across the annotated exons, and a subsequent principal component analysis-based method was used on the observed read counts to differentiate capture kits. This was followed by a hybrid distance- and density-based clustering approach to curate batches of samples to process in parallel. After, the caller was iteratively run for each batch and metrics produced by the Bayesian model were used to account for positive predictive value and sensitivity. GATK-gCNV exome QC filters were previously benchmarked in 8,439 matching genome and exome samples, as described in^54^.

Samples where GATK-gCNV made more than 100 unfiltered calls or more than 10 filtered calls were considered outlier samples and were removed. This resulted in 48,767 samples (~90% of initial) for the downstream burden analysis, which comprises 18,963 epilepsy cases (including 1,743 DEEs, 4,980 GGE, and 8,425 NAFE) and 29,804 controls. To mitigate false positives, we used previously benchmarked filtering thresholds, where CNVs had to span >4 callable exons and had a site frequency <0.1% and a quality score >200. In the gene-based burden analysis of CNVs, we considered CNVs to affect a gene if ≥10% of the non-redundant exon-basepairs overlapped with the deletion (N_gene_= 4,213, 4,417, 4,733, and 6,045 for the analysis of DEEs, GGE, NAFE, and all-epilepsy combined, respectively), or if ≥75% of the non-redundant exon-basepairs overlapped with the duplication (N_gene_= 7,064, 7,282, 7,564, and 8,793 for the analysis of DEEs, GGE, NAFE, and all-epilepsy combined, respectively). When evaluating the joint burden of CNV deletions and protein-truncating SNVs/indels, only the subset of samples passing CNV calling QC were considered.

## Extended Data

## Supplementary Material

Supplement 1

Supplement 2

## Data Availability

We provide summary-level data at the variant and gene level in an online browser for visualization and download (https://epi25.broadinstitute.org/). There are no restrictions on the aggregated data released on the browser. Full results from the exome-wide burden analysis are also available in Supplementary Datasets 1 and 4. WES data from Epi25 cohorts are available via the NHGRI’s controlled-access AnVIL platform (https://anvilproject.org/; dbGaP accession phs001489). Data availability of non-Epi25 control cohorts is provided in the supplementary materials.

## References

[1] Fisher R.S. ILAE official report: a practical clinical definition of epilepsy. Epilepsia 55, 475–82 (2014). 24730690 10.1111/epi.12550

[2] Organization, W.H. Epilepsy: a public health imperative., (2022).

[3] Annegers J.F., Hauser W.A., Anderson V.E. & Kurland L.T. The risks of seizure disorders among relatives of patients with childhood onset epilepsy. Neurology 32, 174–9 (1982). 7198743 10.1212/wnl.32.2.174

[4] Berkovic S.F., Howell R.A., Hay D.A. & Hopper J.L. Epilepsies in twins: genetics of the major epilepsy syndromes. Ann Neurol 43, 435–45 (1998). 9546323 10.1002/ana.410430405

[5] Helbig I., Scheffer I.E., Mulley J.C. & Berkovic S.F. Navigating the channels and beyond: unravelling the genetics of the epilepsies. Lancet Neurol 7, 231–45 (2008). 18275925 10.1016/S1474-4422(08)70039-5

[6] Epi K.C. De novo mutations in epileptic encephalopathies. Nature 501, 217–21 (2013). 23934111 10.1038/nature12439 PMC3773011

[7] Euro, E.-R.E.S.C., Epilepsy Phenome/Genome, P. & Epi K.C. De novo mutations in synaptic transmission genes including DNM1 cause epileptic encephalopathies. Am J Hum Genet 95, 360–70 (2014). 25262651 10.1016/j.ajhg.2014.08.013 PMC4185114

[8] Epi K.C. De Novo Mutations in SLC1A2 and CACNA1A Are Important Causes of Epileptic Encephalopathies. Am J Hum Genet 99, 287–98 (2016). 27476654 10.1016/j.ajhg.2016.06.003 PMC4974067

[9] McTague A., Howell K.B., Cross J.H., Kurian M.A. & Scheffer I.E. The genetic landscape of the epileptic encephalopathies of infancy and childhood. Lancet Neurol 15, 304–16 (2016). 26597089 10.1016/S1474-4422(15)00250-1

[10] Heyne H.O. De novo variants in neurodevelopmental disorders with epilepsy. Nat Genet 50, 1048–1053 (2018). 29942082 10.1038/s41588-018-0143-7

[11] Epi, K.c. & Epilepsy Phenome/Genome, P. Ultra-rare genetic variation in common epilepsies: a case-control sequencing study. Lancet Neurol 16, 135–143 (2017). 28102150 10.1016/S1474-4422(16)30359-3

[12] May P. Rare coding variants in genes encoding GABAA receptors in genetic generalised epilepsies: an exome-based case-control study. Lancet Neurol 17, 699–708 (2018). 30033060 10.1016/S1474-4422(18)30215-1

[13] Epi25 Collaborative. Electronic address, s.b.u.e.a. & Epi, C. Ultra-Rare Genetic Variation in the Epilepsies: A Whole-Exome Sequencing Study of 17,606 Individuals. Am J Hum Genet 105, 267–282 (2019). 31327507 10.1016/j.ajhg.2019.05.020 PMC6698801

[14] Baldassari S. The landscape of epilepsy-related GATOR1 variants. Genet Med 21, 398–408 (2019). 30093711 10.1038/s41436-018-0060-2 PMC6292495

[15] Epi25 Collaborative. Electronic address, j.c.c.e. & Epi, C. Sub-genic intolerance, ClinVar, and the epilepsies: A whole-exome sequencing study of 29,165 individuals. Am J Hum Genet 108, 965–982 (2021). 33932343 10.1016/j.ajhg.2021.04.009 PMC8206159

[16] Samocha K.E. Regional missense constraint improves variant deleteriousness prediction. BioRxiv, 148353 (2017).

[17] Barwell J., Snape K. & Wedderburn S. The new genomic medicine service and implications for patients. Clin Med (Lond) 19, 273–277 (2019). 31308102 10.7861/clinmedicine.19-4-273 PMC6752257

[18] Sirmaci A. Mutations in ANKRD11 cause KBG syndrome, characterized by intellectual disability, skeletal malformations, and macrodontia. Am J Hum Genet 89, 289–94 (2011). 21782149 10.1016/j.ajhg.2011.06.007 PMC3155157

[19] Skjei K.L., Martin M.M. & Slavotinek A.M. KBG syndrome: report of twins, neurological characteristics, and delineation of diagnostic criteria. Am J Med Genet A 143A, 292–300 (2007). 17230487 10.1002/ajmg.a.31597

[20] Low K. Clinical and genetic aspects of KBG syndrome. Am J Med Genet A 170, 2835–2846 (2016). 27667800 10.1002/ajmg.a.37842 PMC5435101

[21] Guo L. KBG syndrome: videoconferencing and use of artificial intelligence driven facial phenotyping in 25 new patients. Eur J Hum Genet (2022). 10.1038/s41431-022-01171-1 PMC9626563 35970914

[22] Dibbens L.M. Mutations in DEPDC5 cause familial focal epilepsy with variable foci. Nat Genet 45, 546–51 (2013). 23542697 10.1038/ng.2599

[23] Ishida S. Mutations of DEPDC5 cause autosomal dominant focal epilepsies. Nat Genet 45, 552–5 (2013). 23542701 10.1038/ng.2601 PMC5010101

[24] Bar-Peled L. A Tumor suppressor complex with GAP activity for the Rag GTPases that signal amino acid sufficiency to mTORC1. Science 340, 1100–6 (2013). 23723238 10.1126/science.1232044 PMC3728654

[25] Baulac S. mTOR signaling pathway genes in focal epilepsies. Prog Brain Res 226, 61–79 (2016). 27323939 10.1016/bs.pbr.2016.04.013

[26] Lal D. DEPDC5 mutations in genetic focal epilepsies of childhood. Ann Neurol 75, 788–92 (2014). 24591017 10.1002/ana.24127

[27] Goodspeed K. Current knowledge of SLC6A1-related neurodevelopmental disorders. Brain Commun 2, fcaa170 (2020). 33241211 10.1093/braincomms/fcaa170 PMC7677605

[28] Absalom N.L. Gain-of-function and loss-of-function GABRB3 variants lead to distinct clinical phenotypes in patients with developmental and epileptic encephalopathies. Nat Commun 13, 1822 (2022). 35383156 10.1038/s41467-022-29280-x PMC8983652

[29] Lal D. Gene family information facilitates variant interpretation and identification of disease-associated genes in neurodevelopmental disorders. Genome Med 12, 28 (2020). 32183904 10.1186/s13073-020-00725-6 PMC7079346

[30] Ruepp A. CORUM: the comprehensive resource of mammalian protein complexes. Nucleic Acids Res 36, D646–50 (2008). 17965090 10.1093/nar/gkm936 PMC2238909

[31] Farrant M. & Nusser Z. Variations on an inhibitory theme: phasic and tonic activation of GABA(A) receptors. Nat Rev Neurosci 6, 215–29 (2005). 15738957 10.1038/nrn1625

[32] Maljevic S. Spectrum of GABAA receptor variants in epilepsy. Curr Opin Neurol 32, 183–190 (2019). 30664068 10.1097/WCO.0000000000000657

[33] Zhu S. Structure of a human synaptic GABAA receptor. Nature 559, 67–72 (2018). 29950725 10.1038/s41586-018-0255-3 PMC6220708

[34] Adzhubei I., Jordan D.M. & Sunyaev S.R. Predicting functional effect of human missense mutations using PolyPhen-2. Curr Protoc Hum Genet Chapter 7, Unit7 20 (2013). 10.1002/0471142905.hg0720s76 PMC4480630 23315928

[35] Vaser R., Adusumalli S., Leng S.N., Sikic M. & Ng P.C. SIFT missense predictions for genomes. Nat Protoc 11, 1–9 (2016). 26633127 10.1038/nprot.2015.123

[36] Kellogg E.H., Leaver-Fay A. & Baker D. Role of conformational sampling in computing mutation-induced changes in protein structure and stability. Proteins 79, 830–8 (2011). 21287615 10.1002/prot.22921 PMC3760476

[37] Hallmann K. A homozygous splice-site mutation in CARS2 is associated with progressive myoclonic epilepsy. Neurology 83, 2183–7 (2014). 25361775 10.1212/WNL.0000000000001055

[38] Coughlin C.R. 2nd Mutations in the mitochondrial cysteinyl-tRNA synthase gene, CARS2, lead to a severe epileptic encephalopathy and complex movement disorder. J Med Genet 52, 532–40 (2015). 25787132 10.1136/jmedgenet-2015-103049

[39] Samanta D., Gokden M. & Willis E. Clinicopathologic Findings of CARS2 Mutation. Pediatr Neurol 87, 65–69 (2018). 30139652 10.1016/j.pediatrneurol.2018.06.009

[40] Kapoor D., Majethia P., Anand A., Shukla A. & Sharma S. Expanding the electro-clinical phenotype of CARS2associated neuroregression. Epilepsy Behav Rep 16, 100485 (2021). 34704010 10.1016/j.ebr.2021.100485 PMC8524140

[41] Kobow K. Deep sequencing reveals increased DNA methylation in chronic rat epilepsy. Acta Neuropathol 126, 741–56 (2013). 24005891 10.1007/s00401-013-1168-8 PMC3825532

[42] Pusalkar M. Acute and Chronic Electroconvulsive Seizures (ECS) Differentially Regulate the Expression of Epigenetic Machinery in the Adult Rat Hippocampus. Int J Neuropsychopharmacol 19(2016). 10.1093/ijnp/pyw040 PMC5043647 27207907

[43] Xu J. MicroRNA expression profiling after recurrent febrile seizures in rat and emerging role of miR-148a-3p/SYNJ1 axis. Sci Rep 11, 1262 (2021). 33441699 10.1038/s41598-020-79543-0 PMC7806659

[44] Berkovic S.F., Cavalleri G.L. & Koeleman B.P. Genome-wide meta-analysis of over 29,000 people with epilepsy reveals 26 loci and subtype-specific genetic architecture. medRxiv, 2022.06.08.22276120 (2022).

[45] Priori S.G. Mutations in the cardiac ryanodine receptor gene (hRyR2) underlie catecholaminergic polymorphic ventricular tachycardia. Circulation 103, 196–200 (2001). 11208676 10.1161/01.cir.103.2.196

[46] Laitinen P.J. Mutations of the cardiac ryanodine receptor (RyR2) gene in familial polymorphic ventricular tachycardia. Circulation 103, 485–90 (2001). 11157710 10.1161/01.cir.103.4.485

[47] Tiso N. Identification of mutations in the cardiac ryanodine receptor gene in families affected with arrhythmogenic right ventricular cardiomyopathy type 2 (ARVD2). Hum Mol Genet 10, 189–94 (2001). 11159936 10.1093/hmg/10.3.189

[48] Meli A.C. A novel ryanodine receptor mutation linked to sudden death increases sensitivity to cytosolic calcium. Circ Res 109, 281–90 (2011). 21659649 10.1161/CIRCRESAHA.111.244970 PMC3690513

[49] Fujii Y. A type 2 ryanodine receptor variant associated with reduced Ca(2+) release and short-coupled torsades de pointes ventricular arrhythmia. Heart Rhythm 14, 98–107 (2017). 27756708 10.1016/j.hrthm.2016.10.015

[50] Cheung J.W. Short-coupled polymorphic ventricular tachycardia at rest linked to a novel ryanodine receptor (RyR2) mutation: leaky RyR2 channels under non-stress conditions. Int J Cardiol 180, 228–36 (2015). 25463374 10.1016/j.ijcard.2014.11.119 PMC4281514

[51] Lehnart S.E. Leaky Ca2+ release channel/ryanodine receptor 2 causes seizures and sudden cardiac death in mice. J Clin Invest 118, 2230–45 (2008). 18483626 10.1172/JCI35346 PMC2381750

[52] Yap S.M. & Smyth S. Ryanodine receptor 2 (RYR2) mutation: A potentially novel neurocardiac calcium channelopathy manifesting as primary generalised epilepsy. Seizure 67, 11–14 (2019). 30849713 10.1016/j.seizure.2019.02.017

[53] Kaplanis J. Evidence for 28 genetic disorders discovered by combining healthcare and research data. Nature 586, 757–762 (2020). 33057194 10.1038/s41586-020-2832-5 PMC7116826

[54] Fu J.M. Rare coding variation provides insight into the genetic architecture and phenotypic context of autism. Nat Genet (2022). 10.1038/s41588-022-01104-0 PMC9653013 35982160

[55] Singh T. Rare coding variants in ten genes confer substantial risk for schizophrenia. Nature 604, 509–516 (2022). 35396579 10.1038/s41586-022-04556-w PMC9805802

[56] Jepsen K. SMRT-mediated repression of an H3K27 demethylase in progression from neural stem cell to neuron. Nature 450, 415–9 (2007). 17928865 10.1038/nature06270

[57] Estaras C. Genome-wide analysis reveals that Smad3 and JMJD3 HDM co-activate the neural developmental program. Development 139, 2681–91 (2012). 22782721 10.1242/dev.078345

[58] Park D.H. Activation of neuronal gene expression by the JMJD3 demethylase is required for postnatal and adult brain neurogenesis. Cell Rep 8, 1290–9 (2014). 25176653 10.1016/j.celrep.2014.07.060 PMC4201382

[59] Shan Y. JMJD3 and UTX determine fidelity and lineage specification of human neural progenitor cells. Nat Commun 11, 382 (2020). 31959746 10.1038/s41467-019-14028-x PMC6971254

[60] Wang W., Cho H., Lee J.W. & Lee S.K. The histone demethylase Kdm6b regulates subtype diversification of mouse spinal motor neurons during development. Nat Commun 13, 958 (2022). 35177643 10.1038/s41467-022-28636-7 PMC8854633

[61] Kruidenier L. A selective jumonji H3K27 demethylase inhibitor modulates the proinflammatory macrophage response. Nature 488, 404–8 (2012). 22842901 10.1038/nature11262 PMC4691848

[62] Moloney P.B., Cavalleri G.L. & Delanty N. Epilepsy in the mTORopathies: opportunities for precision medicine. Brain Commun 3, fcab222 (2021). 34632383 10.1093/braincomms/fcab222 PMC8495134

[63] Gielen M. & Corringer P.J. The dual-gate model for pentameric ligand-gated ion channels activation and desensitization. J Physiol 596, 1873–1902 (2018). 29484660 10.1113/JP275100 PMC5978336

[64] Johannesen K.M. Genotype-phenotype correlations in SCN8A-related disorders reveal prognostic and therapeutic implications. Brain 145, 2991–3009 (2022). 34431999 10.1093/brain/awab321 PMC10147326

[65] Oyrer J. Ion Channels in Genetic Epilepsy: From Genes and Mechanisms to Disease-Targeted Therapies. Pharmacol Rev 70, 142–173 (2018). 29263209 10.1124/pr.117.014456 PMC5738717

[66] Syrbe S. De novo loss- or gain-of-function mutations in KCNA2 cause epileptic encephalopathy. Nat Genet 47, 393–399 (2015). 25751627 10.1038/ng.3239 PMC4380508

[67] Sheikh B.N., Guhathakurta S. & Akhtar A. The non-specific lethal (NSL) complex at the crossroads of transcriptional control and cellular homeostasis. EMBO Rep 20, e47630 (2019). 31267707 10.15252/embr.201847630 PMC6607013

[68] Koolen D.A. Mutations in the chromatin modifier gene KANSL1 cause the 17q21.31 microdeletion syndrome. Nat Genet 44, 639–41 (2012). 22544363 10.1038/ng.2262

[69] Zollino M. Mutations in KANSL1 cause the 17q21.31 microdeletion syndrome phenotype. Nat Genet 44, 636–8 (2012). 22544367 10.1038/ng.2257

[70] Miller N., Lacroix E.M. & Backus J.E. MEDLINEplus: building and maintaining the National Library of Medicine′s consumer health Web service. Bull Med Libr Assoc 88, 11–7 (2000). 10658959 PMC35193

[71] Koolen D.A. Clinical and molecular delineation of the 17q21.31 microdeletion syndrome. J Med Genet 45, 710–20 (2008). 18628315 10.1136/jmg.2008.058701 PMC3071570

[72] Tan T.Y. Phenotypic expansion and further characterisation of the 17q21.31 microdeletion syndrome. J Med Genet 46, 480–9 (2009). 19447831 10.1136/jmg.2008.065391

[73] Fujiwara K. Deletion of JMJD2B in neurons leads to defective spine maturation, hyperactive behavior and memory deficits in mouse. Transl Psychiatry 6, e766 (2016). 27023172 10.1038/tp.2016.31 PMC4872455

[74] Duncan A.R. Heterozygous Variants in KDM4B Lead to Global Developmental Delay and Neuroanatomical Defects. Am J Hum Genet 107, 1170–1177 (2020). 33232677 10.1016/j.ajhg.2020.11.001 PMC7820620

[75] Specchio N. & Curatolo P. Developmental and epileptic encephalopathies: what we do and do not know. Brain 144, 32–43 (2021). 33279965 10.1093/brain/awaa371

[76] Azevedo M.F. Clinical and molecular genetics of the phosphodiesterases (PDEs). Endocr Rev 35, 195–233 (2014). 24311737 10.1210/er.2013-1053 PMC3963262

[77] Delhaye S. & Bardoni B. Role of phosphodiesterases in the pathophysiology of neurodevelopmental disorders. Mol Psychiatry 26, 4570–4582 (2021). 33414502 10.1038/s41380-020-00997-9 PMC8589663

[78] Erro R., Mencacci N.E. & Bhatia K.P. The Emerging Role of Phosphodiesterases in Movement Disorders. Mov Disord 36, 2225–2243 (2021). 34155691 10.1002/mds.28686 PMC8596847

[79] Lee D. Global and local missions of cAMP signaling in neural plasticity, learning, and memory. Front Pharmacol 6, 161 (2015). 26300775 10.3389/fphar.2015.00161 PMC4523784

[80] Threlfell S. & West A.R. Review: Modulation of striatal neuron activity by cyclic nucleotide signaling and phosphodiesterase inhibition. Basal Ganglia 3, 137–146 (2013). 24490129 10.1016/j.baga.2013.08.001 PMC3904398

[81] Zhang Y. The Phosphodiesterase 10A Inhibitor PF-2545920 Enhances Hippocampal Excitability and Seizure Activity Involving the Upregulation of GluA1 and NR2A in Post-synaptic Densities. Front Mol Neurosci 10, 100 (2017). 28439226 10.3389/fnmol.2017.00100 PMC5383654

[82] Carhuapoma J.R., Qureshi A.I., Tamargo R.J., Mathis J.M. & Hanley D.F. Intra-arterial papaverine-induced seizures: case report and review of the literature. Surg Neurol 56, 159–63 (2001). 11597640 10.1016/s0090-3019(01)00450-5

[83] Koko M. Distinct gene-set burden patterns underlie common generalized and focal epilepsies. EBioMedicine 72, 103588 (2021). 34571366 10.1016/j.ebiom.2021.103588 PMC8479647

[84] Wolff M. Genetic and phenotypic heterogeneity suggest therapeutic implications in SCN2A-related disorders. Brain 140, 1316–1336 (2017). 28379373 10.1093/brain/awx054

[85] Brunklaus A. Biological concepts in human sodium channel epilepsies and their relevance in clinical practice. Epilepsia 61, 387–399 (2020). 32090326 10.1111/epi.16438

[86] Brunklaus A. SCN1A variants from bench to bedside-improved clinical prediction from functional characterization. Hum Mutat 41, 363–374 (2020). 31782251 10.1002/humu.23943

[87] Masnada S. Clinical spectrum and genotype-phenotype associations of KCNA2-related encephalopathies. Brain 140, 2337–2354 (2017). 29050392 10.1093/brain/awx184

[88] Malerba F. Genotype-phenotype correlations in patients with de novo KCNQ2 pathogenic variants. Neurol Genet 6, e528 (2020). 33659638 10.1212/NXG.0000000000000528 PMC7803337

[89] Harris P.A. Research electronic data capture (REDCap)--a metadata-driven methodology and workflow process for providing translational research informatics support. J Biomed Inform 42, 377–81 (2009). 18929686 10.1016/j.jbi.2008.08.010 PMC2700030

[90] Collaborative E. The epilepsy phenome/genome project. Clin Trials 10, 568–86 (2013).23818435

[91] Van der Auwera G.A. From FastQ data to high confidence variant calls: the Genome Analysis Toolkit best practices pipeline. Curr Protoc Bioinformatics 43, 11 10 1–11 10 33 (2013). 10.1002/0471250953.bi1110s43 PMC4243306 25431634

[92] McLaren W. The Ensembl Variant Effect Predictor. Genome Biol 17, 122 (2016). 27268795 10.1186/s13059-016-0974-4 PMC4893825

[93] Karczewski K.J. The mutational constraint spectrum quantified from variation in 141,456 humans. Nature 581, 434–443 (2020). 32461654 10.1038/s41586-020-2308-7 PMC7334197

[94] Team H. Hail. 0.2.62-84fa81b9ea3d. https://github.com/hail-is/hail/commit/84fa81b9ea3d.edn.

[95] Li H. Toward better understanding of artifacts in variant calling from high-coverage samples. Bioinformatics 30, 2843–51 (2014). 24974202 10.1093/bioinformatics/btu356 PMC4271055

[96] Babadi M. GATK-gCNV: A Rare Copy Number Variant Discovery Algorithm and Its Application to Exome Sequencing in the UK Biobank. bioRxiv, 2022.08.25.504851 (2022).

